# The Nature of a Writing System Shapes the Cognitive and Neural Mechanisms for Reading Acquisition

**DOI:** 10.1162/NOL.a.248

**Published:** 2026-05-21

**Authors:** J. S. H. Taylor, Adam Jowett, Tibor Auer, Cheng-Yu Hsieh, Angelika Lingnau, Kathleen Rastle

**Affiliations:** Division of Psychology and Language Sciences, University College London, London, UK; Department of Psychology, Royal Holloway, University of London, Egham, UK; Advanced Research Computing Centre, University College London, London, UK; Faculty of Human Science, University of Regensburg, Regensburg, Germany

**Keywords:** artificial orthography, neuroimaging, reading acquisition, reading development, statistical regularities

## Abstract

Writing systems are cultural inventions that differ in how they represent spoken language. We tested how the brain learns to map arbitrary visual symbols to sound and meaning by comparing neural activity for artificial writing systems that were either alphabetic (systematic symbol-sound mappings) or logographic (arbitrary symbol-sound mappings). Twenty-four adults learned to read aloud and comprehend novel words written in each system. After 2 weeks of training, functional magnetic resonance imaging during reading comprehension revealed that the dorsal pathway (inferior parietal gyrus, inferior frontal gyrus), which is involved in mapping from print-to-sound, was more active for the alphabetic system, whereas the ventral pathway (anterior fusiform gyrus, middle temporal gyrus), which is involved in mapping from print-to-meaning, was more active for the logographic system. Combined with performance differences, these findings indicate that systematic symbol-sound mappings allowed the brain to bridge the interface between vision and meaning via sound, whereas an absence of systematicity made it more efficient to link vision directly to meaning. Thus, the same brain finds different solutions to the problem of reading that capitalise on the statistical properties of culturally invented writing systems.

## INTRODUCTION

The central challenge of reading acquisition is learning to transform meaningless visual symbols into meaningful spoken language. Writing systems are cultural inventions that vary substantially in how they represent spoken language. Thus, the same human brain must be able to learn the mapping between vision and language for any of the world’s writing systems, but research has yet to discover how this is achieved. We tested the hypothesis that the brain finds different solutions to the problem of reading acquisition that are governed by statistical regularities in the writing system being learned (Seidenberg, 2011).

Thousands of years of cultural evolution have produced writing systems in which the mapping from vision to language ranges from individual symbols representing individual sounds to complex characters representing whole concepts. The same brain must be able to find an efficient solution to mapping written to spoken language that works for all of these writing systems. In computational simulations, statistical regularities in the writing system influence the “division of labour” between two reading pathways during learning (Harm & Seidenberg, 2004; Plaut et al., 1996; Smith et al., 2021). One such regularity is whether visual symbols relate systematically to sounds, as is the case in alphabetic systems (such as Spanish) but not in logographic systems (of which Chinese is the closest example). A pathway from print-to-sound-to-meaning is more heavily used when the model learns to read alphabetic as compared to logographic systems (Smith et al., 2021). Conversely, a direct pathway from print-to-meaning should be preferred where there is no symbol-sound systematicity since this is a more efficient way to comprehend text (Seidenberg, 2011).

**Figure 1.**
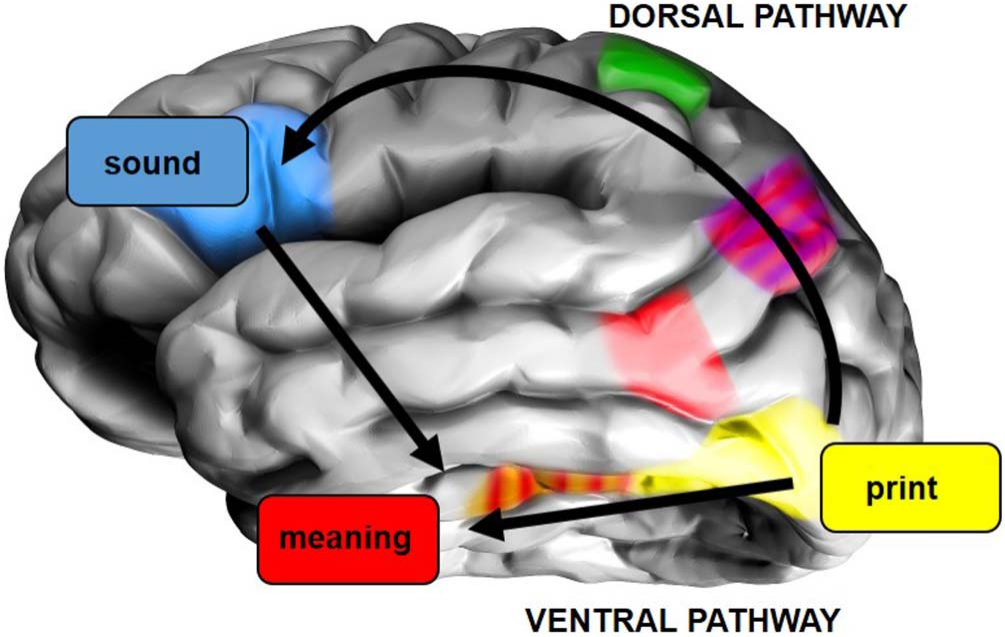
Dorsal and ventral pathways for reading, adapted from Taylor et al. (2013). Written word forms are processed in posterior occipitotemporal cortex (yellow area). Within the dorsal pathway, inferior/superior parietal gyri (green area) are involved in mapping from print to sound and dorsal inferior frontal gyrus (blue area) in generating a spoken output. Within the ventral pathway, anterior fusiform gyrus (red/orange striped area) processes whole-word written forms and/or their meanings and middle temporal gyrus (red area) processes word meanings. The red/purple striped area is angular gyrus, which is not a core component of either pathway.

Empirical evidence for this division of labour cannot come from behavioural data, such as response times for different writing systems, since these do not directly diagnose pathway reliance (Seidenberg, 2011). Such evidence could be provided by neuroimaging data since the two reading pathways are associated with distinct brain regions (Figure 1). Left dorsal regions (inferior/superior parietal gyri, dorsal inferior frontal gyrus) support print-to-sound mapping, whereas, left ventral regions (anterior fusiform gyrus, middle temporal gyrus) support print-to-meaning mapping (Carreiras et al., 2014; Cattinelli et al., 2013; McNorgan et al., 2015; Price, 2012; Taylor et al., 2013). Combining this with a framework for relating computational models to neural activity (Taylor et al., 2013), dorsal pathway regions should be more engaged when learning to comprehend alphabetic systems, since there should be greater reliance on mapping from print-to-sound and then to meaning, whereas ventral pathway regions should be more engaged when learning to comprehend logographic systems, since there should be greater reliance on direct print-to-meaning mapping.

Unfortunately, studies comparing neural activity when participants read in different languages are difficult to interpret because of multiple confounds between languages, writing systems, and participants. For example, one study of bilingual adults found that English versus Chinese word reading activated dorsal versus ventral reading pathways, respectively (Dong et al., 2020), but this may have been confounded with proficiency, which was higher for Chinese. Between-group (Rueckl et al., 2015) and meta-analytic (Zhang et al., 2024) comparisons of skilled readers in fact find minimal differences in brain activity between alphabetic versus logographic systems. Finally, though a meta-analysis (Zhang et al., 2024) provides some indication that these differences may be more pronounced in children, consistent differences were only observed in one direction (Chinese > English), again suggesting possible confounds with proficiency and writing system complexity. Furthermore, the comparison between English and Chinese as examples of alphabetic versus logographic systems is flawed. English is an opaque alphabetic system with many words that do not conform to regular symbol-sound rules, and Chinese characters contain phonetic radicals that convey (relatively inconsistent) pronunciation information and semantic radicals that convey (somewhat more consistent) meaning information.

Artificial orthographies can be designed to test how specific differences between writing systems impact the division of labour between reading pathways in the early stages of learning to read. They also provide total control over participants’ prior knowledge. Alphabetic artificial orthographies activate the same neural reading pathways as natural languages (Taylor et al., 2014a, 2017, 2019), with dorsal versus ventral pathways engaged for learning novel word pronunciations versus meanings, respectively (Taylor et al., 2017). There are hints from such studies that different writing systems engage different reading pathways, but results may have been influenced by participants’ native language. Specifically, in English native speakers, dorsal pathway activity (left supramarginal gyrus) was observed for reading a newly learned alphabetic relative to a logographic script, but the reverse comparison highlighted only right hemisphere regions (Mei et al., 2014). Conversely, in Chinese native speakers, ventral pathway activity (bilateral middle temporal gyrus) was observed for reading a logographic relative to an alphabetic script, but no regions were identified by the reverse comparison (Mei et al., 2015). Furthermore, these studies were likely underpowered due to the use of between-subject designs and did not look at reading comprehension since participants were not taught meanings for the novel words.

We tested whether the same participants learning to comprehend an alphabetic versus a logographic writing system would show differential engagement of the dorsal and ventral reading pathways. Participants learned to read a set of novel words written in a transparent alphabetic system, which had an entirely systematic mapping between symbols and sounds, and a set of words written in a logographic system, in which these mappings were entirely arbitrary. All other aspects of the language, writing system, and teaching method were controlled. After two weeks of training, participants completed a reading comprehension task whilst neural activity was measured with functional magnetic resonance imaging (fMRI). We predicted that the dorsal pathway would be more engaged when reading the alphabetic script, whereas the ventral pathway would be more engaged when reading the logographic script. We also expected that, for behavioural tasks conducted outside of the scanner, performance would be superior for the alphabetic system on reading aloud, which prioritises print-to-sound mapping, but for the logographic system on reading comprehension, which prioritises print-to-meaning mapping.

## METHODS

### Participants

Twenty-four monolingual native English-speaking adults (16 females) aged 19–35 (*M* = 22.16, *SD* = 3.97) who worked or studied at Royal Holloway, University of London, UK (RHUL), participated for £150. Participants were right-handed, with no history of hearing impairment, uncorrected visual impairment, or learning difficulties. All participants gave informed consent and satisfied exclusion criteria and safety requirements for MRI scanning. Royal Holloway University of London ethics committee approved the research.

### Stimuli (Examples in Figure 2)

#### Phonological forms

Two sets of 24 consonant–vowel–consonant pseudowords were recorded by a native English female speaker (digitised at a sampling rate of 44.1 kHz). Pseudowords were constructed from eight consonants, /b/, /f/, /g/, /m/, /p/, /t/, /v/, /z/, and eight vowels, four of which were used for each pseudoword set; /ɛ/, /ʌ/, /aɪ/, /əʊ/ (set 1); /æ/, /ɒ/, /i/, /u/, (set 2). Within each set, consonants occurred three times in onset position and three times in coda position, whereas vowels occurred six times each. Two additional sets of pseudowords were constructed to serve as untrained items in recognition memory post-test tasks (not reported here) by replacing one consonant or one vowel from a trained item. Spoken and written forms of all stimuli are shared on the open science framework (https://osf.io/nujm3/overview).

**Figure 2.**
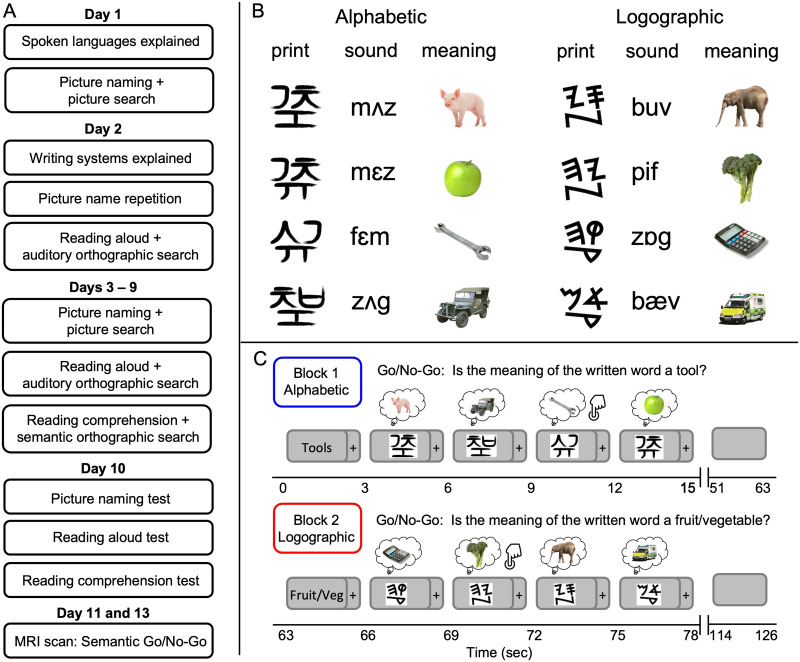
Panel A shows the tasks completed on each day of training and testing for which data are reported in the current manuscript. Note that across Days 3–9 the tasks were completed in different orders and the starting writing system for each task alternated each day. Panel B presents example stimuli to illustrate the properties of each writing system. Panel C shows the in-scanner reading comprehension task (Semantic Go/No-Go) in which participants read and covertly retrieved the meanings of the trained words. Alphabetic and logographic systems were presented in alternating blocks (starting system counterbalanced across participants). A semantic category cue at the start of each block instructed participants to press a button on the MRI-compatible button box if the written word corresponded to the semantic category.

#### Semantic forms

Two sets of 24 familiar objects were selected from the Hemera Photo Objects 50,000 Premium Image collection (or the internet, *N* = 3). Each set comprised six fruits or vegetables, six vehicles, six animals, and six tools. Each set of meanings was associated with one pseudoword set, but, within each set, the meaning to spoken form assignment was counterbalanced across participants such that half received one assignment and half another. Copyright prevents us from sharing these pictures.

#### Written forms

Each participant learned one pseudoword set written in symbols taken from Korean Hangul and one written in symbols taken from an archaic Phoenician alphabet, with the three symbols comprising each written form arranged as a triangle. For each participant, one set was assigned to be Alphabetic (phonemes associated with symbols in a one-to-one manner), and one set was assigned to be Logographic (phonemes associated with symbols arbitrarily). The assignment of pseudoword set to Korean/Phoenician and to Alphabetic/Logographic was counterbalanced across participants.

### Behavioural Training and Testing Procedure (Overview in Figure 2)

On each day, training took ≤1.5 hr and all tasks were self-paced. Task order rotated each day. For each training and test task, 12 participants began with the alphabetic script and 12 began with the logographic script and this alternated across days. Within each task, items were presented in a randomised order. All tasks were presented using E-Prime (version 2.0; Psychology Software Tools, 2003). For tasks with spoken responses, there was a 9-s time limit on each trial and responses were recorded and manually coded for accuracy and RT. Keyboard and mouse accuracy and RTs were recorded by E-Prime.

#### Day 1

Participants were first taught that each pseudoword that they would learn had a consonant–vowel–consonant structure, that each had a meaning from one of four semantic categories, and that meanings were arbitrarily associated with pseudoword pronunciations (see Training slides 1, https://osf.io/nujm3/files/u5jvz). They then completed the following tasks for each pseudoword set.

##### Picture name repetition.

Participants saw a picture of each meaning, heard its associated pseudoword, and repeated it aloud (three blocks of 24 trials per set). Since this was a simple repetition task, results are not reported.

##### Picture naming.

Participants saw a picture of each meaning, said aloud the associated pseudoword, and pressed the spacebar to hear the correct answer (three blocks of 24 trials per set).

##### Picture search.

This task was included to reinforce learning of sound-to-meaning mappings. Participants heard a trained pseudoword and used the mouse to click on the associated picture from a 4 × 6 grid of all 24 meanings. The correct item then appeared in a green circle (three blocks of 24 trials per set, grid organised differently on each block).

##### English language tasks.

English spelling and vocabulary were measured as part of a wider project (Bathelt et al., 2024; Sagi et al., 2024, 2025) and are not reported. In the spelling task, participants heard an English word, first in isolation and then in a sentence, and typed its spelling. The 40 items were taken from Burt and Tate (2002; see also Ulicheva, Coltheart, et al., 2021; Ulicheva, Marelli, et al., 2021) and were 8–10 letters and low frequency. They could press backspace to change their response. There was no time limit and they pressed enter to move on to the next trial. In the vocabulary task, participants saw a target word and below it four other words from which they had to select the one closest in meaning to the target. The 40 items were taken from Shipley (1940) and increased in difficulty across the task.

#### Day 2

Participants were taught about the structure of the two scripts (see Training Slides 2, https://osf.io/nujm3/files/95ktd). They were first taught about the alphabetic script (Language 1). They were told that there was a one-to-one mapping between symbols and sounds and that the consonants appeared at the top of each word with the vowel underneath. They were also shown three words differing only in one symbol and one sound as examples. They then completed the three tasks described below for the alphabetic script. Once training for the alphabetic script was finished, participants received instruction on the structure of the logographic script (Language 2). They were told that there was no relationship between the symbols in a word and its pronunciation and again were shown three words that differed only in one symbol but in multiple sounds as examples. Participants then completed the below tasks for the logographic script.

##### Picture name repetition with written form.

On each trial participants saw the written form and picture for a trained pseudoword, heard its pronunciation, and repeated it aloud (one block of 24 trials per set).

##### Reading aloud.

Participants saw the written form of a trained pseudoword, said aloud its pronunciation, and pressed the spacebar to hear the correct answer (three blocks of 24 trials per set).

##### Auditory orthographic search.

This task was included to reinforce learning of print-to-sound mappings. Participants heard a trained pseudoword and used the mouse to click on the associated written form from a 4 × 6 grid of all 24 trained items. The correct item then appeared in a green circle (one block of 24 trials per set).

#### Days 3 to 9

##### Picture naming (Days 3, 5, 7, 9).

As on Day 1, but with only one block per set.

##### Picture search (Days 4, 6, 8).

As on Day 1, but with only one block per set.

##### Reading aloud (all Days).

As on Day 2.

##### Auditory orthographic search (all Days).

As on Day 2.

##### Reading comprehension (all Days).

Participants saw the written form of a trained pseudoword, said aloud its meaning, and pressed spacebar to hear the correct answer (three blocks of 24 trials per set).

##### Semantic orthographic search (all Days).

This task was included to reinforce learning of the print-to-meaning mappings. Participants saw a picture of one of the trained pseudoword meanings and used the mouse to click on the associated written form from a four-by-size grid of all 24 trained items. The correct item then appeared in a green circle (one block of 24 trials per set).

#### Day 10

##### Picture naming test.

As on Days 3–9 but no feedback was provided.

##### Reading aloud test.

As on Days 2–9. Due to experimenter error, three blocks for each script were completed, and feedback was provided. However, the results are only reported for the first block.

##### Reading comprehension test.

As on Days 3–9 but only one block was completed for each script and no feedback was provided.

##### Visual recognition memory.

Participants made judgements as to whether a written form was one they had learned (trained item, press Z) or not learned (untrained, press M). Each trained item target was presented twice and there were 48 untrained distractors. This task was included as part of a wider project and results are therefore not reported.

##### Semantic go/no-go task (practice for MRI scan).

Participants saw the written forms of trained pseudowords and had to judge whether they were from a cued semantic category. There were four blocks of six trials for each script with a target semantic category displayed at the beginning of each block. On each trial, participants pressed the spacebar if the meaning of the written pseudoword corresponded with the target category (time limit of 3 s). No feedback was given, but at the end of block 4 they either saw “Well Done! It looks like you’re concentrating on the task” if they responded during any of the trials or “Oops! Try and concentrate! You didn’t respond to any of the trials” if they did not. Results are not reported for this practice task.

##### Auditory test tasks.

Several auditory only tasks were included in the test battery as part of a wider project (results are therefore not reported). In auditory shadowing, participants listened to and repeated each trained pseudoword. In phoneme reversal, participants listened to each trained pseudoword and repeated it but swapped the onset and coda consonants (e.g., /bεv/ should be repeated as /vεb/). In auditory recognition memory, participants made judgements as to whether a spoken form was one they had learned (trained item, press Z) or not learned (untrained, press M). Each trained item target was presented twice and there were 48 untrained distractors. In auditory semantic go/no-go, participants practiced the auditory task they completed in the scanner. The task was the same as the visual semantic go/no-go task but involved listening (rather than reading) trained pseudowords.

### Behavioural Data Analyses

Two tasks focused on sound–meaning mapping (picture naming, picture search), two focused on print–sound mapping (reading aloud, auditory orthographic search), and two focused on print–meaning mapping (reading comprehension, semantic orthographic search). Results for the first task in each pair are reported in the main manuscript; results for the second are reported on the OSF along with all behavioural data and analysis scripts (https://osf.io/nujm3/overview). Linear Mixed-Effects (LME) models were constructed to assess performance on each training task as a function of the fixed factors Script and Day, and each test task as a function of the fixed factor Script. In training data analyses, Day was mean-centered (cDay) as continuous fixed factors should be standardised to manage collinearity and to aid interpretation of resulting coefficients (Meteyard & Davies, 2020). Accuracy and RTs were modelled using Binomial (logistic) and Gaussian (linear) distributions, respectively, using the lme4 (Version 1.1.34; Bates et al., 2015), and lmerTest (Version: 3.1.3; Kuznetsova et al., 2017) packages in R (Version 4.3.0; R Core Team, 2021). RT data were log transformed (logRT) to reduce distribution skewing. All data points were included in accuracy analyses while RT analyses only included correct responses. No outliers were removed from the training data. For the test data, histograms were plotted to review the distribution of RT data and outliers were removed (prior to the calculation of condition means) as follows: Picture Naming > 3,999 ms, Reading aloud > 6,999 ms, Reading Comprehension > 7,999 ms, Semantic Go/No-Go < 500 ms. For the semantic go/no-go task, only correct Go trials were included in RT analyses.

LME models included participant (Subject) and semantic form (Sem) as random factors. The random effects model specified intercepts only. The contrast for the fixed factor of Script was specified using deviation coding, or sum-to-zero coding (alphabetic: 0.5, logographic: −0.5). Under this scheme, positive regression coefficients (*b*) indicate larger values for the alphabetic condition, whereas negative regression coefficients (*b*) indicate larger values for the logographic condition. Model equations were as follows:

Accuracy during training: glmer(Acc ∼ Script * cDay + (1|Subject) + (1|Sem), data = results, family = binomial)
RT during training: lmer(logRT ∼ Script * cDay + (1|Subject) + (1|Sem), data = correct)
Accuracy at test: glmer(Acc ∼ Script + (1|Subject) + (1|Sem), data = results, family = binomial)
RT at test: lmer(logRT ∼ Script + (1|Subject) + (1|Sem), data = correct).

The regression coefficient (*b*), odds ratio (*OR*), standard error (*SE*), *Z*, and *p* values are reported for LME models analysing accuracy data. *b*, *SE*, *t*, and *p* values are reported for RT models. *b* is the unstandardised fixed-effect coefficient (the estimated difference between conditions having controlled for random effects) and is logit transformed for accuracy models. *OR* is derived from *b* and is the log transformed ratio of the probability of responding accurately for the alphabetic condition over the probability of responding accurately for the logographic condition. Test statistics *Z* and *t* denote the estimated effect size having accounted for random effects and variability in the dataset (i.e., *SE*). *Z* is reported for generalised linear models as they do not assume the dependent variable is normally distributed (i.e., binomial accuracy data), while *t* is reported for linear models as they assume the dependent variable is normally distributed (i.e., continuous logRT data).

For the in-scanner semantic go/no-go task, accuracy data were not analysed using LME models and instead paired samples *t*-tests were conducted to examine the effect of Script on d-prime values.

### MRI Scanning Procedure (Figure 2)

#### In-scanner task

Scanning took place on Days 11 and 13 with two runs of a reading comprehension task (visual semantic go/no-go) completed on each day. These alternated with two auditory semantic go/no-go runs (half the participants began with auditory, half with visual), the results of which are not reported since they are not the focus of the current study. Each run comprised 12 blocks of 16 trials, with blocks alternating between the alphabetic and logographic script (beginning script counterbalanced across participants). Each trained item was presented four times per run. Each block began with presentation of a target semantic category, with each category used three times per run in a randomised order. Each block finished with 12 s of rest (blank screen) but after every third block this was instead a feedback screen saying either “Well done! It looks like you’re concentrating on the task”, if they responded on any trial, or “Oops! Try and concentrate! You didn’t respond to any of the trials”, if they did not. Visual stimuli were projected onto a screen behind the scanner bore and viewed using a mirror positioned on the head coil. Auditory stimuli were delivered using high quality etymotic earphones (Sensimetrics model S14). On each trial, participants saw or heard a trained item and were instructed to press a button with their right index finger on an MRI compatible button box (NATA Technologies) if the meaning matched the target semantic category within 3 s. Responses were recorded using E-Prime (version 2.0; Psychology Software Tools, 2003).

#### Scanning parameters

Structural and functional MRI data were obtained using a 3T Siemens Trio scanner (Siemens Medical Systems, Erlangen, Germany) and a 32-channel head coil. High resolution T1-weighted structural images were obtained using a Magnetisation Prepared Rapid Acquisition Gradient Echo sequence with the following acquisition parameters: 1 mm slice thickness, 1 mm isotropic resolution, repetition time (TR) = 1,830 ms, echo time (TE) = 3.03 ms, inversion time = 1,100 ms, flip angle (FA) = 11 deg, field of view (FOV) = 256 × 160 × 256 mm. Functional images were acquired with a T2*- weighted gradient echo echoplanar imaging (EPI) sequence optimised for blood oxygenation level dependent contrast. 32 consecutive axial slices were obtained in descending order using the following acquisition parameters: 3 mm slice thickness, 75 mm (25%) interslice gap, 3 mm in-plane resolution, TE = 30 ms, FA = 78°, FOV = 192 mm, 64 × 64 data matrix. The acquisition volume was angled to cover the whole brain, avoiding the eyes and including the cerebellum, but failed to cover the top of the parietal lobe for some participants. Six dummy scans were included at the beginning of each run and excluded from subsequent analyses.

For visual semantic go/no-go, we used a continuous imaging design (TR = 2,000 ms, TA = 2,000 ms) and presented written pseudowords for 2,500 ms followed by a 500 ms fixation cross per trial. Overall, 382 images were acquired during each 12.7-min run. For auditory semantic go/no-go (results not reported), we used a sparse imaging design (TR = 3,000 ms, TA = 2,000 ms) and presented one spoken pseudoword between volume acquisitions (Edmister et al., 1999). Spoken pseudowords were presented with a fixation cross that remained on screen for the duration of the trial. Acquisition was synchronised with E-Prime using transistor-transistor logic pulse inputs from the MRI scanner to ensure each acquisition began 1,000 ms after trial onset. Overall, 258 images were acquired during each 12.9-min run.

### MRI Analyses for Visual Semantic Go/No-Go

#### Pre-processing pipeline

The acquired data were converted into Brain Imaging Data Structure format (BIDS; Gorgolewski et al., 2016). These data are shared on OpenNeuro (doi:10.18112/openneuro.ds006021.v1.0.1). Functional and structural image pre-processing and analysis was achieved using MATLAB (MathWorks) and Automatic Analysis (aa version 5.0.0, Cusack et al., 2015) integrating functions from SPM12 (Wellcome Trust Centre for Functional Neuroimaging, London, www.fil.ion.ucl.ac.uk/spm). Structural images were aligned to Montreal Neurological Institute (MNI) space, corrected for intensity bias, segmented into grey and white matters, and normalised to MNI space using Diffeomorphic Anatomical Registration Through Exponentiated Lie Algebra (Ashburner, 2007).

After removing the first six dummy volumes, EPI images were then spatially (rigid-body) and temporally realigned to the middle image and the middle slice, respectively, in the series to correct for head movement and varying slice acquisition times (Friston et al., 1995). Realigned EPI images were co-registered to each participant’s structural image (Ashburner & Friston, 1997) and normalised to MNI space using the affine and the warping parameters used to normalise their structural image. Normalised EPI images were then resampled to 3 mm isotropic voxels and spatially filtered using a 5 mm full width at half maximum isotropic Gaussian Kernel. Finally, a grey matter mask was created for each participant and co-registered to the EPI images. Masks included voxels exceeding 10% probability of being grey matter (segmentation threshold = 0.1).

#### Data exclusions

Following pre-processing, three runs from two participants were excluded from analyses as scan-to-scan movement >2 mm was detected during motion correction. A single block from one run for one participant was also marked for exclusion as they admitted to falling asleep. All blocks with no responses were re-reviewed for movement associated with sleep, but none needed to be excluded. No run needed to be removed due to poor behavioural performance as it was consistently high.

#### First and second level analyses

Fully pre-processed data for the four runs from each participant were entered into a general linear model analysis (within-subject design). Box-car functions representing events associated with each writing system were convolved with the canonical Haemodynamic Response Function (HRF) within SPM12 to create one regressor per writing system in each run. For each run, semantic category targets at the beginning of each block were explained away as a regressor of no interest, convolved with the HRF. One block of one run for one participant that was marked for exclusion due to sleep was also explained away using a covariate (i.e., not convolved with HRF). Motion correction parameters estimated during the spatial realignment stage of pre-processing were included as covariates. Finally, rest served as an implicit baseline for subsequent contrasts. A high-pass temporal filter of 128 s was applied to remove low-frequency signal drifts.

Voxel-wise contrasts identified activation for alphabetic and logographic trained items relative to rest (alphabetic > baseline, logographic > baseline), and for alphabetic relative to logographic trained items (alphabetic > logographic) and vice versa (logographic > alphabetic).

Contrasts of parameter estimates were entered into group-level one-sample *t*-tests to compare first-level observations to the null hypothesis with participants as a random effect. Any voxels located outside MNI space following normalisation were removed. Group-level results were thresholded at voxel-wise *p* < .001 (uncorrected), while only activations greater than a cluster-extent family wise error (FWE) threshold of *p* < .05 (corrected) were considered significant.

Regions of interest (ROI) analyses were conducted using the MarsBaR toolbox (Brett et al., 2002) for the contrast of alphabetic versus logographic trained items. Left hemisphere ROIs were defined based on a meta-analysis of fMRI and PET studies comparing activity for word versus pseudoword reading (Taylor et al., 2013). As proposed in that meta-analysis, regions showing greater activity for pseudoword than word reading likely play a role in print-to-sound mapping whereas regions showing greater activity for word than pseudoword reading likely play a role in print-to-meaning mapping. Therefore, two dorsal pathway ROIs that showed [pseudoword > word] activity were defined in inferior frontal gyrus and inferior parietal gyrus, and two ventral pathway ROIs that showed [word > pseudoword] activity were defined in anterior fusiform and middle temporal gyrus. The ROIs included the full extent of the original clusters identified in the meta-analysis (details are provided in Table 1).

**Table 1.** Regions of interest analyses comparing activation for trained words from the alphabetic versus logographic writing systems during a reading comprehension task (semantic go/no-go).

Label	*x*	*y*	*z*	Volume (mm)	*t*-stat	*p* value	*η* ^2^
inferior frontal/precentral gyrus	−46	6	26	12616	12.46	<.001	18.86
inferior parietal gyrus	−36	−48	46	952	4.75	<.001	10.29
parahippocampal/anterior fusiform gyrus	−32	−36	−12	1512	−3.66	<.001	−3.54
middle temporal gyrus	−64	−54	−10	824	−2.83	.005	−4.70

*Note*. ROIs were defined based on Taylor et al. (2013) and co-ordinates refer to the top peak in the cluster. Labels refer to regions within the cluster and are based on the automated anatomical labelling template. *p*-values are Bonferroni corrected for four comparisons. *η*^2^ is the effect size (contrast estimate given in MarsBaR).

## RESULTS

### Neural Activity During Reading Comprehension

#### Reading both writing systems engages dorsal and ventral reading pathways

**Figure 3.**
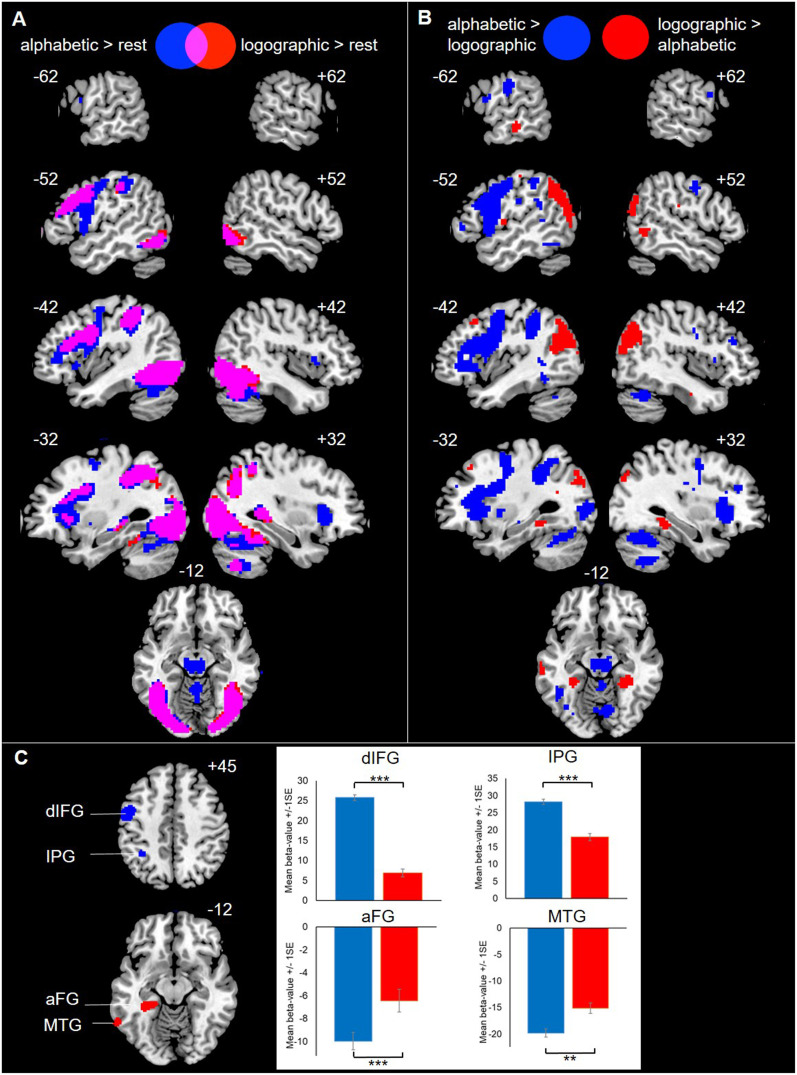
Whole-brain and region of interest analyses comparing activation for trained words from the alphabetic versus logographic writing systems during a reading comprehension task (semantic go/no-go). Panel A shows activation for the alphabetic (blue) and logographic (red) systems, relative to the implicit resting baseline, with overlap shown in pink, *p* < .001 uncorrected, *p* < .05 FWE cluster extent corrected (Supplementary Material Tables 1 and 2). Panel B shows activation that was greater for the alphabetic than the logographic system (blue) or for the logographic than the alphabetic system (red). Note that these results are shown at an uncorrected threshold of *p* < .001 in order to show left ventral clusters, but the majority of clusters are significant at *p* < .05 FWE cluster extent corrected (Supplementary Materials Tables 3 and 4). Panel C shows activation for the alphabetic (blue) and logographic (red) system, relative to the implicit resting baseline, in the regions of interest given in Table 1. dIFG = dorsal inferior frontal gyrus, IPG = inferior parietal gyrus, aFG = anterior fusiform gyrus, MTG = middle temporal gyrus.

Relative to the implicit resting baseline, alphabetic and logographic systems both activated bilateral occipitotemporal and parietal cortices, left inferior frontal and precentral gyri, and bilateral supplementary motor areas (Supplementary Materials Tables 1 and 2, Figure 3; Supporting Information can be found at https://doi.org/10.1162/NOL.a.248). This mirrors previous work (Taylor et al., 2017) in demonstrating that reading in artificial orthographies activates similar regions to reading words in existing alphabetic writing systems (Taylor et al., 2013). It also establishes that any differences in pathway use between the two systems are relative, since both activate the same reading network. Accuracy on the in-scanner reading comprehension task (Supplementary Material Figure 1) was extremely high for both the alphabetic (d-prime = 3.06, *SE* = .06) and the logographic (d-prime = 3.50, *SE* = .06) script, though higher for the latter (*t*(23) = −3.82, *p* < .001). RTs on Go trials were also faster for the logographic (*M* = 1,326 ms, *SE* = 13.9) than the alphabetic (*M* = 1,747 ms, *SE* = 13.9) script (*b* = 0.29, *SE* = 0.02, *t* = 13.00, *p* < .001).

#### Alphabetic and logographic systems preferentially engage dorsal versus ventral reading pathways, respectively

Activity was greater for the alphabetic than the logographic system in bilateral inferior and superior parietal gyri extending to supramarginal gyrus, left inferior frontal and precentral gyri, left posterior occipitotemporal cortex, and the cerebellum. In contrast, activity was greater for the logographic than the alphabetic system in bilateral angular gyri extending to middle occipital and middle temporal gyrus, precuneus, left middle frontal gyrus, and right parahippocampal gyrus. When the statistical threshold was lowered from the original *p* < .05 FWE cluster extent corrected to *p* < .001 uncorrected, a 30-voxel cluster in left anterior fusiform/parahippocampal gyrus and a 24-voxel cluster in left anterior middle temporal gyrus also showed greater activity for the logographic than the alphabetic system (Supplementary Materials Tables 3 and 4, Figure 3 Panels B and C).

These results were confirmed with regions of interest analyses (Table 1, Figure 3 Panel C). Inferior frontal gyrus and inferior parietal gyrus ROIs showed greater activity for the alphabetic than the logographic system. In contrast, anterior fusiform gyrus and middle temporal gyrus ROIs showed the reverse pattern, although it should be noted that these ventral pathway regions were deactive relative to rest for both writing systems, an issue we return to in the Discussion. Overall, these findings support the hypothesis that the division of labour between the dorsal and ventral reading pathways early in learning depends on the systematicity of print-to-sound mappings.

### Behavioural Performance

#### Sound-to-meaning mapping is equivalent for the two systems

Performance on the picture-naming task was examined to establish that this was equivalent for the two writing systems (Supplementary Material Figure 2). Training data analyses showed that accuracy increased (*b* = 0.32, *SE* = 0.01, *Z* = 26.63, *p* < .001, *OR* = 1.38) and response time (RT) decreased (*b* = −0.06, *SE* < 0.01, *t* = −28.44, *p* < .001) across training sessions. There was no main effect of script (Accuracy: *b* = 0.12, *SE* = 0.26, *Z* = 0.45, *p* = .655, *OR* = 1.13; RT: *b* = −0.04, *SE* = 0.04, *t* = −0.93, *p* = .354), though there was an interaction between session and script in accuracy (*b* = 0.06, *SE* = 0.02, *Z* = 2.37, *p* = .018, *OR* = 1.06), but not RT (*b* = −0.01, *SE* < 0.01, *t* = −1.33, *p* = .182), reflecting somewhat greater accuracy for the alphabetic script on Days 5 and 7. A very similar pattern was observed in the picture search task (results reported on the OSF). At test, picture naming accuracy was extremely high (Alphabetic mean proportion correct = 0.98, *SE* = .01; Logographic mean proportion correct = 0.98, *SE* = .01) and did not differ between the two scripts (*b* = −0.01, *SE* = 0.52, *Z* = −0.01, *p* = .991, *OR* = 0.99). RTs were also equivalent for the two scripts (Alphabetic mean RT = 1,190 ms, *SE* = 17 ms; Logographic mean RT = 1,136 ms, *SE* = 17 ms; *b* = 0.03, *SE* = 0.04, *t* = 0.93, *p* = .355). Overall, the results of the sound–meaning mapping tasks suggest no difference in performance between the two writing systems, which was as expected since this mapping was arbitrary in both systems.

#### Print-to-sound mapping is more accurate and faster for the alphabetic than the logographic system

**Figure 4.**
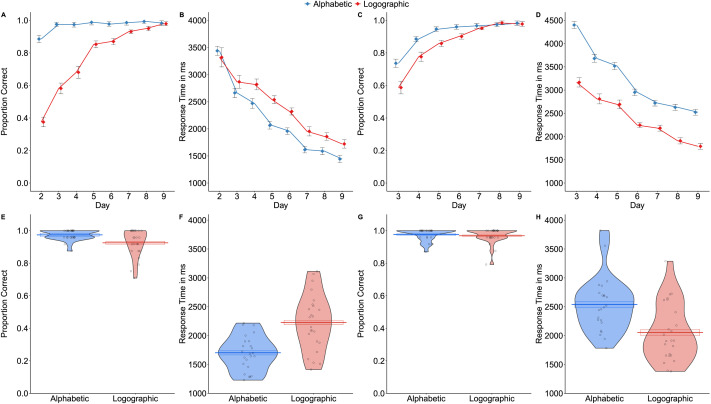
Reading aloud (A, B, E, F) and reading comprehension (C, D, G, H) performance for the two writing systems. A and C show mean proportion correct during training. B and D show mean response time for correct items during training. E and G show proportion correct at test. F and H show response times for correct items at test. In A–D means are calculated across participants and error bars reflect the standard error adjusted for the within-participant design. In E–H, centre lines show the mean across participants, boxes around this line show the standard error adjusted for the within-participant design, dots represent individual participants, and the violins reflect the shape of the distribution across participants.

Performance on the reading aloud task during training and at test is shown in Figure 4. Accuracy was higher (*b* = 2.23, *SE* = 0.22, *Z* = 10.02, *p* < .001, *OR* = 9.26) and RTs were faster (*b* = −0.11, *SE* = 0.03, *t* = −3.96, *p* < .001) for the alphabetic than the logographic system. Accuracy increased (*b* = 0.56, *SE* = 0.03, *Z* = 20.89, *p* < .001, *OR* = 1.74) and RTs decreased (*b* = −0.11, *SE* < 0.01, *t* = −53.13, *p* < .001) across training sessions, though improvement was much slower for the logographic than the alphabetic system (session x script interaction; Accuracy: *b* = −0.34, *SE* = 0.05, *Z* = −6.37, *p* < .001, *OR* = 0.71, RT: *b* = −0.02, *SE* < 0.01, *t* = −3.77, *p* < .001). The same pattern of performance was also observed on the auditory orthographic search task (results reported on the OSF). Reading aloud performance remained superior for the alphabetic system at test (Accuracy: *b* = 1.14, *SE* = 0.33, *Z* = 3.41, *p* = .001, *OR* = 3.13; RT: *b* = −0.24, *SE* = 0.03, *t* = −7.98, *p* < .001). These results converge with the neuroimaging data in suggesting that print-to-sound systematicity supports the use of this mapping, which benefits reading aloud accuracy and speed.

#### Print-to-meaning mapping is faster for the logographic than the alphabetic system

Performance on the reading comprehension task during training and at test is shown in Figure 4. Accuracy was higher for the alphabetic than the logographic system (*b* = 0.65, *SE* = 0.22, *Z* = 2.98, *p* = .003, *OR* = 1.91) and increased over training (*b* = 0.68, *SE* = 0.03, *Z* = 24.42, *p* < .001, *OR* = 1.98), though this improvement was somewhat slower for the logographic than the alphabetic system (session × system interaction: *b* = −0.11, *SE* = 0.05, *Z* = −1.98, *p* = .048, *OR* = .90). In contrast, RTs were faster for the logographic system throughout training (*b* = 0.34, *SE* = 0.04, *t* = 9.50, *p* < .001). They also decreased over training for both systems (main effect of session: *b* = −0.10, *SE* < 0.01, *t* = −41.45, *p* < .001, no session × system interaction: *b* < 0.01, *SE* < 0.01, *t* = 0.49, *p* = .623). A similar pattern of performance was observed on the semantic orthographic search task, with higher accuracy for the alphabetic system early in training but faster RTs for the logographic system throughout training (results reported on the OSF). At test, reading comprehension accuracy did not differ for the two systems (*b* = 0.29, *SE* = 0.48, *Z* = 0.61, *p* = .541, *OR* = 1.34), but RTs were faster for the logographic than the alphabetic system (*b* = 0.26, *SE* = 0.04, *t* = 6.80, *p* < .001). Faster RTs for the logographic than the alphabetic system during training and at test suggest that participants were more reliant on mapping directly from print-to-meaning rather than from print-to-sound-to-meaning for the former than the latter. These results again converge with the neuroimaging data and suggest that arbitrary print-to-sound mappings lead to a greater reliance on the print-to-meaning pathway during reading comprehension.

## DISCUSSION

Our results demonstrate that the same brain finds different solutions to building an interface between vision and language depending on the statistical regularities of the writing system being learned (Smith et al., 2021). During reading comprehension, the alphabetic and logographic systems showed overlapping activation across the typical reading network (Rueckl et al., 2015; Taylor et al., 2017, 2019). Yet, the statistical regularities characterising these systems influenced the relative engagement of the two pathways in this network within the same participants. The dorsal pathway was more engaged when participants read words written in the alphabetic than the logographic system, whereas the ventral pathway was more engaged when reading the logographic than the alphabetic system. Thus, when print-to-sound mappings were completely systematic (alphabetic system), the brain capitalised on this and relied more on mapping from visual to spoken forms and then using sound-to-meaning knowledge to comprehend the words. Conversely, in the absence of print-to-sound systematicity (logographic system), it was more efficient for the brain to rely on mapping directly from vision to meaning.

Within the dorsal pathway, there is consensus that left inferior frontal and precentral gyri are involved in generating the sounds of words during reading (in this case covertly) (Carreiras et al., 2014; Price, 2012; Taylor et al., 2013). With respect to inferior/superior parietal gyrus activation, some have argued that this reflects the visual attention demands of reading (Cohen et al., 2008). However, counterbalancing ensured that these demands were equivalent for our two systems. Our results therefore support a more specific role for inferior parietal gyrus in mapping visual to spoken forms in a componential manner (i.e., letter-by-letter, Taylor et al., 2013, 2014a), which was viable for the alphabetic but not the logographic system.

Within the ventral pathway, bilateral anterior fusiform and parahippocampal gyri and left middle temporal gyrus have all been implicated in semantic processing (Binder et al., 2009; Taylor et al., 2013). However, left anterior fusiform gyrus may instead represent whole-word written forms (Taylor et al., 2013) or be an interface between written forms and semantics (Purcell et al., 2014). These latter explanations may be a better fit for our data since the in-scanner task required word meaning to be accessed for both systems, what differed was the greater reliance on direct visual form-to-meaning mapping for the logographic system. A somewhat unexpected aspect of our ventral pathway findings was that both left middle temporal and anterior fusiform gyri were deactivated relative to rest for both writing systems, though to a lesser extent for the logographic system. Several authors have proposed that participants engage in semantic processing when at rest (Binder, 2012; Seghier, 2013). We therefore suggest that reading the logographic system engaged the semantic processes supported by these regions more than the alphabetic system and thus activity deviated to a lesser extent from the “semantic resting state”. However, this requires further investigation.

Several regions outside the dorsal and ventral pathways were differentially activated by the two scripts and deserve discussion. A cluster in left posterior occipitotemporal cortex, overlapping with the canonical visual word form area (VWFA), was more active for the alphabetic than the logographic system. This result is consistent with the view that the VWFA represents letters and letter clusters, rather than whole-word forms (Dehaene et al., 2005; Vinckier et al., 2007), since words in the alphabetic system could be read letter-by-letter, whereas words in the logographic system were more likely read as wholes. Both right and left cerebellum (primarily lobule 6) were also more active for the alphabetic than the logographic script. These posterior cerebellar regions have been implicated in phonological and orthographic processing in previous neuroimaging studies of reading (Li et al., 2022). Clusters in bilateral angular gyri were more active for the logographic than the alphabetic script but, similar to the ventral pathway regions, were deactivated relative to rest for both writing systems. It may be that reading in the logographic writing system resulted in less deactivation because it engaged some of the semantic processes that these regions have been proposed to carry out during the resting state (Binder et al., 2009; Seghier, 2013). However, as angular gyri are also part of the default mode network (Buckner et al., 2008; Gusnard & Raichle, 2001), it is difficult to draw firm conclusions about their role in reading (see Graves et al., 2017, 2023, for further discussion; Seghier, 2013; Taylor et al., 2013, 2014b).

We now consider how the division of labour between reading pathways may change over development. A previous study involving skilled readers of English and Chinese found no evidence for a division of labour between dorsal and ventral reading pathways in reading comprehension based on writing system (Rueckl et al., 2015). Since these writing systems differ in many ways and comparisons were between-subjects in this previous study, the absence of significant differences may reflect low power. However, a more interesting possibility is that differences in neural activity between writing systems may be less pronounced (or even absent) during skilled reading. One influential theory proposes that readers use print-to-sound systematicity to “self-teach” unfamiliar words, with this “provid[ing] opportunities to learn word-specific print-to-meaning connections” (Share, 1995, p. 151). Therefore, even for writing systems with systematic mappings between print and sound, it becomes more efficient to comprehend written words using direct print-to-meaning connections (see also Smith et al., 2021). This idea is exemplified by performance differences between our two systems. Though reading aloud was more accurate and faster for the alphabetic than the logographic system, reading comprehension was faster for the logographic system, reflecting greater reliance on the direct print-to-meaning mapping. We therefore predict that differences between writing systems should be magnified during learning but decline as readers build their efficiency in transforming visual symbols directly to meaning, which could indeed lead to a “universal brain signature of proficient reading” (Rueckl et al., 2015, p. 15510).

In conclusion, our study demonstrates that systematic symbol-sound mappings allow the brain to bridge the interface between vision and meaning via sound, while an absence of systematicity means that linking vision directly to meaning is more efficient. Thus, the brain finds different solutions to mapping from vision to spoken language depending on the statistical regularities of the writing system being learned. Whilst our study shows how the biology-culture interface operates in real-time, it is also possible that this interface has shaped the evolution of writing systems. Spoken language can be transcribed in a variety of ways but properties of the brain may have ensured that writing systems that are easier to learn and comprehend were favoured (Carr & Rastle, 2024). Understanding the requirements and evolution of how writing systems map onto spoken language can thus cast new light on bidirectional relationships between biological and cultural systems.

## ACKNOWLEDGMENTS

These data were collected whilst Adam Jowett was a PhD student at Royal Holloway, University of London.

## FUNDING INFORMATION

Kathleen Rastle, Economic and Social Research Council (https://dx.doi.org/10.13039/501100000269), Award ID: ES/L002264/1.

## AUTHOR CONTRIBUTIONS

**J. S. H. Taylor**: Conceptualization: Supporting; Data curation: Lead; Formal analysis: Supporting; Funding acquisition: Supporting; Methodology: Supporting; Visualization: Supporting; Writing – original: Equal; Writing – review & editing: Equal. **Adam Jowett**: Conceptualization: Equal; Data curation: Supporting; Formal analysis: Equal; Investigation: Lead; Methodology: Equal; Visualization: Equal; Writing – review & editing: Supporting. **Tibor Auer**: Conceptualization: Supporting; Data curation: Supporting; Formal analysis: Equal; Methodology: Supporting; Visualization: Equal; Writing – review & editing: Supporting. **Cheng-Yu Hsieh**: Data curation: Supporting; Formal analysis: Equal; Visualization: Equal; Writing – review & editing: Supporting. **Angelika Lingnau**: Conceptualization: Supporting; Formal analysis: Supporting; Methodology: Supporting; Visualization: Supporting; Writing – review & editing: Supporting. **Kathleen Rastle**: Conceptualization: Equal; Data curation: Supporting; Formal analysis: Supporting; Funding acquisition: Lead; Investigation: Supporting; Methodology: Equal; Visualization: Supporting; Writing – original: Equal; Writing – review & editing: Equal.

## DATA AND CODE AVAILABILITY STATEMENTS

Stimuli, behavioural data, the behavioural data analysis scripts, depiction of the neuroimaging analysis pipeline, and regions of interest used for neuroimaging analyses are available on the Open Science Framework (https://osf.io/nujm3/overview).

Raw neuroimaging data in BIDS format are available on OpenNeuro (doi:10.18112/openneuro.ds006021.v1.0.1).

## Supplementary Material



## TECHNICAL TERMS

Dorsal pathway: Brain areas involved in mapping from print-to-sound, including superior and inferior parietal gyrus and the dorsal part of inferior frontal gyrus.
Ventral pathway: Brain areas involved in mapping from print-to-meaning, including anterior fusiform gyrus and middle temporal gyrus.
Artificial orthography: An invented writing system comprising unfamiliar symbols.
Reading comprehension: The process of mapping from printed words to their meanings.
Alphabetic writing system: A writing system that uses symbols to represent phonemes.
Logographic writing system: A writing system that uses symbols to represent whole words or morphemes (the meaningful units that comprise words, e.g., un-account-able).
Reading aloud: The process of mapping from printed words to their pronunciations.
Semantics: The meanings of words.
Phonology: The sounds (phonemes) that comprise spoken words.
Orthography: The symbols used to write words, for example letters in the Latin alphabet or characters in Chinese.
