# Supplementary material for: The Nature of a Writing System Shapes the Cognitive and Neural Mechanisms for Reading Acquisition
Source: Neurobiol Lang (Camb). 2026 May 21;7:NOL.a.248. doi: 10.1162/NOL.a.248 (PMC13200069; doi:10.1162/NOL.a.248)
Supplement: Supplementary file 1 [file nol-07-248-s001.pdf]

Supplemental Results

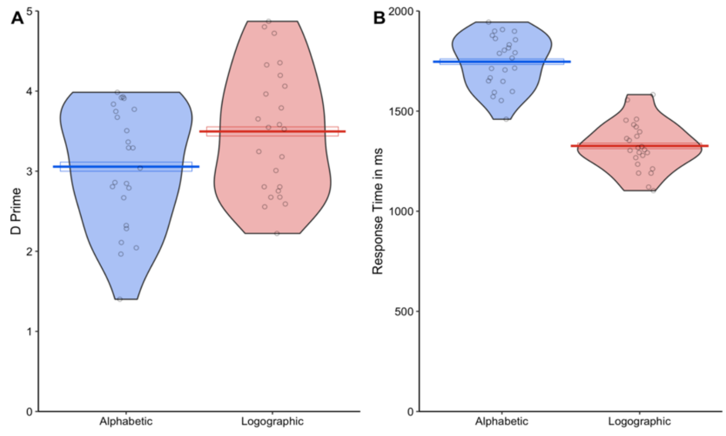

Supplemental Figure 1. Performance on the semantic go/no-go task during fMRI scanning for the two scripts. A. D-prime scores. B. Response time for correct Go trials. Means are calculated across participants and error bars reflect the standard error adjusted for the within-participant design. Centre lines show the mean across participants, boxes around this line show the standard error adjusted for the within-participant design, dots represent individual participants, and the violins reflect the shape of the distribution across participants.

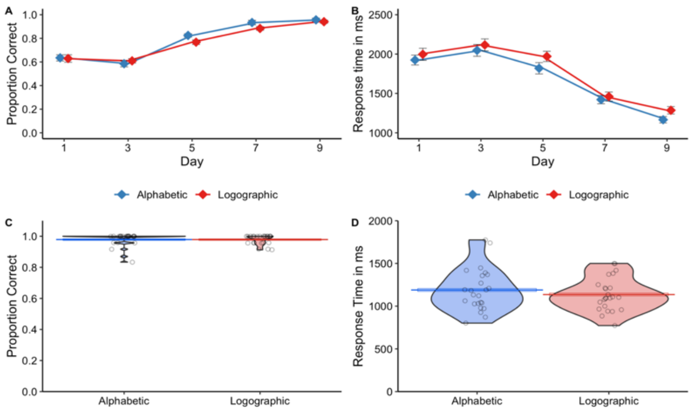

Supplemental Figure 2. Picture naming performance for the two scripts. A. Mean proportion correct during training. B. Mean response time for correct items during training. C. Proportion correct at test. D. Response time for correct items at test. In A and B, means are calculated across participants and error bars reflect the standard error adjusted for the within-participant design. In C and D, centre lines show the mean across participants, boxes around this line show the standard error adjusted for the within-participant design, dots represent individual participants, and the violins reflect the shape of the distribution across participants.

Supplemental Table 1

Activation during reading comprehension for trained words from the alphabetic script, relative to rest.

				MNI co-ordinates		
Region Label	Extent	*t*-value	Cluster *p*-value	x	y	z
**Calcarine_R**	**4451**	**20.329**	**< .001**	**21**	**-90**	**-3**
Occipital_Inf_R				36	-84	-9
Occipital_Inf_R				39	-66	-12
Occipital_Mid_R				30	-90	9
Occipital_Mid_R				33	-87	0
Occipital_Sup_R				24	-99	9
Cerebellum_Crus2_R				6	-78	-33
Undefined				-18	-3	15
Cerebellum_6_R				30	-60	-27
Cerebellum_Crus1_R				39	-66	-27
Undefined				-6	-24	-9
Precentral_L				-42	3	33
Thalamus_L				-9	-18	6
Frontal_Inf_Tri_L				-45	24	24
Vermis_6				6	-75	-18
Undefined				6	-24	-9
Putamen_L				-21	6	3
Vermis_9				3	-54	-30
Cerebellum_8_R				15	-72	-45
Precentral_L				-45	6	21
**Occipital_Inf_L**	**2082**	**15.119**	**< .001**	**-33**	**-87**	**-9**
Occipital_Mid_L				-21	-90	-3
Occipital_Inf_L				-15	-96	-9
Occipital_Mid_L				-27	-90	6
Occipital_Mid_L				-30	-84	0

Occipital_Inf_L				-39	-72	-6
Lingual_L				-24	-93	-15
Temporal_Inf_L				-48	-51	-12
Parietal_Inf_L				-42	-39	45
Parietal_Sup_L				-24	-66	48
Occipital_Mid_L				-24	-66	36
Undefined				-42	-45	-9
Parietal_Inf_L				-30	-45	45
Fusiform_L				-36	-81	-15
Cerebellum_6_L				-36	-66	-24
Occipital_Mid_L				-30	-78	24
Fusiform_L				-39	-39	-21
Cerebellum_6_L				-30	-57	-27
Cerebellum_Crus1_L				-42	-57	-30
**Hippocampus_R**	**143**	**12.272**	**< .001**	**27**	**-36**	**6**
Undefined				24	-27	-3
Undefined				21	-27	21
**Supp_Motor_Area_L**	**339**	**11.994**	**< .001**	**3**	**12**	**48**
Supp_Motor_Area_L				-6	12	51
**Occipital_Sup_R**	**308**	**8.418**	**< .001**	**27**	**-66**	**45**
Occipital_Mid_R				30	-63	36
Parietal_Inf_R				30	-48	51
**Insula_R**	**121**	**6.205**	**< .001**	**33**	**27**	**0**
Frontal_Inf_Oper_R				42	15	6

Note. Top 20 peaks > 8mm apart are reported at a threshold of $p < .001$ uncorrected, and $p < .05$ FWE cluster corrected. Bold text denotes the first peak within a cluster, hemisphere is given by _L or _R. Anatomical labels in this and all subsequent tables were generated using the automated anatomical labelling template (Rolls et al., 2015) implemented in the bsmpview toolbox (Spunt, 2016) within SPM12.

Supplemental Table 2

Activation during reading comprehension for trained words from the logographic script, relative to rest.

				MNI co-ordinates		
Region Label	Extent	*t*-value	cluster *p*-value	x	y	z
**Occipital_Inf_R**	**1227**	**22.894**	**< .001**	**27**	**-93**	**-3**
Occipital_Mid_R				33	-87	0
Occipital_Inf_R				39	-81	-9
Occipital_Inf_R				39	-66	-12
Fusiform_R				36	-54	-12
Fusiform_R				33	-42	-21
Cerebellum_Crus1_R				39	-66	-30
Cerebellum_6_R				27	-60	-27
**Occipital_Inf_L**	**1717**	**17.060**	**< .001**	**-24**	**-90**	**-6**
Occipital_Mid_L				-21	-93	3
Occipital_Inf_L				-18	-96	-9
Occipital_Inf_L				-36	-81	-6
Occipital_Inf_L				-42	-69	-6
Occipital_Mid_L				-27	-87	3
Occipital_Inf_L				-45	-63	-12
Temporal_Mid_L				-42	-60	-3
Occipital_Mid_L				-27	-69	36
Parietal_Sup_L				-27	-60	51
Parietal_Inf_L				-42	-42	51
Fusiform_L				-39	-48	-12
Parietal_Inf_L				-33	-45	54
Parietal_Inf_L				-45	-27	42
Occipital_Mid_L				-30	-84	24
Fusiform_L				-36	-39	-24
**Occipital_Sup_R**	**313**	**8.955**	**< .001**	**27**	**-66**	**45**

Occipital_Mid_R				30	-66	33
Undefined				27	-57	42
Parietal_Sup_R				30	-48	48
**Hippocampus_R**	**96**	**8.520**	**.001**	**30**	**-36**	**3**
Undefined				24	-27	-3
**Cerebellum_Crus2_R**	**309**	**8.456**	**< .001**	**9**	**-81**	**-30**
Vermis_9				0	-54	-30
Cerebellum_8_R				30	-66	-48
Undefined				18	-63	-39
Undefined				-15	-60	-36
**Precentral_L**	**417**	**8.392**	**< .001**	**-42**	**6**	**33**
Frontal_Inf_Tri_L				-42	24	21
Frontal_Inf_Tri_L				-36	15	30
Precentral_L				-54	3	42
Frontal_Mid_2_L				-48	42	15
**Supp_Motor_Area_R**	**147**	**8.225**	**< .001**	**6**	**15**	**48**
Supp_Motor_Area_L				-6	15	48
**Undefined**	**53**	**7.076**	**.024**	**-30**	**-33**	**0**
**Insula_L**	**51**	**6.493**	**.028**	**-30**	**24**	**3**

Note. Top 20 peaks > 8mm apart are reported at a threshold of $p < .001$ uncorrected, and $p < .05$ FWE cluster corrected. Bold text denotes the first peak within a cluster, hemisphere is given by _L or _R.

Supplemental Table 3

Activation during reading comprehension that was greater for trained words from the alphabetic than the logographic script.

				MNI co-ordinates		
Region Label	Extent	*t*-value	Cluster *p*-value	x	y	z
**Cerebellum_6_R**	**1903**	**12.349**	**< .001**	**30**	**-63**	**-24**
Cerebellum_8_R				18	-72	-48
Cerebellum_8_R				30	-60	-54
Parietal_Inf_L				-27	-45	45
Cerebellum_Crus1_R				45	-57	-30
Cerebellum_6_R				9	-72	-18
Parietal_Inf_L				-42	-36	42
Parietal_Sup_L				-21	-57	51
Postcentral_L				-60	-15	27
Occipital_Sup_L				-24	-78	21
Occipital_Mid_L				-27	-87	12
Parietal_Inf_L				-27	-51	54
SupraMarginal_L				-45	-36	33
Cerebellum_Crus2_L				3	-81	-30
Temporal_Sup_L				-57	-42	21
Lingual_L				-3	-66	-3
Occipital_Inf_L				-27	-81	-6
SupraMarginal_L				-57	-30	30
Vermis_9				0	-54	-33
Calcarine_R				15	-75	15
**Frontal_Inf_Oper_L**	**3886**	**11.110**	**< .001**	**-48**	**9**	**21**
Precentral_L				-45	-3	48
Supp_Motor_Area_R				3	9	57
Frontal_Inf_Oper_L				-45	9	6

Frontal_Inf_Oper_L				-54	9	3
Frontal_Sup_2_L				-27	-6	51
Thalamus_L				-12	-15	9
Undefined				-24	21	9
Undefined				-21	-6	18
Frontal_Inf_Tri_L				-45	18	24
Postcentral_L				-54	-6	42
Insula_L				-36	21	3
Frontal_Inf_Tri_L				-33	24	12
Putamen_L				-21	0	6
Undefined				-12	-6	-6
Frontal_Inf_Tri_L				-36	30	3
Thalamus_L				-6	-6	3
Cingulate_Mid_R				9	15	42
Frontal_Inf_Tri_L				-48	39	3
Undefined				-33	15	18
**Undefined**	**15**	**5.431**	**.629**	**-15**	**18**	**21**
**Cerebellum_6_L**	**119**	**5.190**	**.001**	**-27**	**-60**	**-21**
Cerebellum_6_L				-30	-51	-33
Fusiform_L				-30	-75	-15
Cerebellum_Crus1_L				-42	-60	-30
Cerebellum_6_L				-18	-60	-15
Lingual_L				-21	-78	-15
Cerebellum_6_L				-21	-69	-18
Cerebellum_6_L				-18	-57	-27
**Frontal_Sup_2_R**	**30**	**4.601**	**.228**	**27**	**51**	**-3**
**Undefined**	**22**	**4.586**	**.399**	**-45**	**-45**	**6**
**Temporal_Inf_L**	**53**	**4.570**	**.047**	**-48**	**-57**	**-15**
Temporal_Inf_L				-48	-48	-15
Undefined				-42	-48	-6
**Postcentral_R**	**19**	**4.439**	**.489**	**24**	**-51**	**54**

Note. Top 20 peaks > 8mm apart are reported at a threshold of $p < .001$ uncorrected, cluster-level FWE corrected *p*-value also reported. Clusters ≤ 10 voxels are not reported. Bold text denotes the first peak within a cluster, hemisphere is given by _L or _R.

Supplemental Table 4

Activation during reading comprehension that was greater for trained words from the logographic than the alphabetic script.

				MNI co-ordinates		
Region Label	Extent	*t*-value	Cluster *p*-value	x	y	z
**Occipital_Mid_L**	**444**	**9.206**	**< .001**	**-42**	**-72**	**33**
Occipital_Mid_L				-51	-72	15
Occipital_Mid_L				-36	-84	27
Angular_L				-39	-72	45
Angular_L				-45	-63	42
Parietal_Inf_L				-48	-54	51
Temporal_Mid_L				-45	-63	18
Angular_L				-57	-60	24
**Occipital_Mid_R**	**231**	**6.643**	**< .001**	**45**	**-72**	**24**
Occipital_Mid_R				42	-78	30
Occipital_Mid_R				42	-81	21
**ParaHippocampal_L**	**30**	**6.217**	**.228**	**-30**	**-42**	**-9**
**Precuneus_L**	**310**	**6.205**	**< . 001**	**-6**	**-57**	**30**
Cingulate_Mid_L				-12	-39	36
Precuneus_L				0	-60	24
Precuneus_R				18	-54	21
Precuneus_R				12	-51	12
**Frontal_Mid_2_L**	**138**	**5.813**	**< .001**	**-30**	**33**	**48**
Frontal_Sup_2_L				-18	42	48
Frontal_Sup_2_L				-15	27	60
Frontal_Mid_2_L				-42	21	48
Frontal_Mid_2_L				-24	21	57
Frontal_Sup_2_L				-9	48	48
Frontal_Sup_2_L				-21	51	36
Frontal_Mid_2_L				-36	18	54

Frontal_Sup_2_L				-9	54	39
**Frontal_Sup_Medial_L**	**38**	**5.508**	**.130**	**-6**	**60**	**15**
Frontal_Sup_Medial_L				-6	54	24
Frontal_Sup_Medial_L				-9	51	9
**Temporal_Mid_L**	**24**	**5.347**	**.347**	**-66**	**-24**	**-12**
**ParaHippocampal_R**	**60**	**5.128**	**.030**	**33**	**-39**	**-12**
**Temporal_Mid_R**	**48**	**4.876**	**.066**	**57**	**-57**	**0**

Note. Top 20 peaks > 8mm apart are reported at a threshold of $p < .001$ uncorrected, cluster-level FWE corrected *p*-value also reported. Clusters ≤ 10 voxels are not reported. Bold text denotes the first peak within a cluster, hemisphere is given by _L or _R.

References

Rolls, E. T., Joliot, M., & Tzourio-Mazoyer, N. (2015). Implementation of a new parcellation of the orbitofrontal cortex in the automated anatomical labeling atlas. *NeuroImage*, *122*, 1-5. https://doi.org/https://doi.org/10.1016/j.neuroimage.2015.07.075

Spunt, B. (2016). spunt/bspmview: BSPMVIEW (Version 20161108). Zenodo. https://doi.org/https://doi.org/10.5281/zenodo.168074
